# Manual Insertion of Cochlear Implant Electrodes Versus Robot-Assisted Insertion and Analysis by Micro-CT: A Temporal Bone Study

**DOI:** 10.3390/audiolres16020051

**Published:** 2026-03-26

**Authors:** Alexandre Karkas, Clément Arnold, Yann Lelonge, Norbert Laroche, Fabien Tinquaut, Florian Bergandi, Hubert Marotte, Kelly Daouda

**Affiliations:** 1 Department of Otorhinolaryngology—Head & Neck Surgery, University Medical Center of Saint-Etienne, 42000 Saint-Etienne, France; clement.ra.arnold@gmail.com (C.A.); yann.lelonge@chu-st-etienne.fr (Y.L.); kelly.daouda@chu-st-etienne.fr (K.D.); 2 Medical School, Jean Monnet University, 42270 Saint-Priest-en-Jarez, France; hubert.marotte@chu-st-etienne.fr; 3 Research Laboratory SAINBIOSE Inserm U1059, 42000 Saint-Etienne, France; norbert.laroche@univ-st-etienne.fr; 4 Anatomical Laboratory, Medical School, Jean Monnet University, 42000 Saint-Etienne, France; florian.bergandi@univ-st-etienne.fr; 5 Department of Public Health and Statistics, University Medical Center of Saint-Etienne, 42000 Saint-Etienne, France; fabien.tinquaut@chu-st-etienne.fr; 6 Department of Rheumatology, University Medical Center of Saint-Etienne, 42270 Saint-Priest-en-Jarez, France

**Keywords:** cochlear implantation, atraumatic, manual electrode insertion, robotic electrode insertion, RobOtol^®^, cochlear trauma, complete insertion

## Abstract

**Background/Objectives**: Atraumatic electrode array insertion should be targeted in cochlear implantation. Robotic insertion is used in many centers worldwide. Our objective was to evaluate manual electrode placement and robot-assisted placement using RobOtol^®^ on human temporal bones (TBs), in terms of endocochlear trauma and completion of insertion. **Methods**: Sixteen TBs originating from eight bodies were implanted with Medel-FLEX24 electrodes through the round window. The right TB was implanted manually, while the left TB of the same body was implanted using RobOtol^®^ for electrode insertion. Results were analyzed through micro-computed tomography imaging. No statistical analysis was used, given the small sample size; a descriptive interpretation of micro-CT scans was rather preferred. **Results**: In the “manual group”, there were two cases (25%) of insertion trauma: elevation of basilar membrane at basal turn (Eshraghi-stage-1). In the “robotic group”, there were two cases (25%) of insertion trauma: one case of elevation of basilar membrane at the middle turn (Eshraghi-stage-1) and one case of dislocation of all electrodes in scala vestibuli (Eshraghi-stage-3). There were six cases (75%) of incomplete insertion in the “manual group” and four cases (50%) of incomplete insertion in the “robotic group”. **Conclusions**: Both techniques of electrode placement yielded fairly similar results, in terms of endocochlear trauma and completion of insertion. New larger-scale cadaveric and clinical studies are needed to determine the possible benefit of robot-assisted electrode insertion in cochlear implantation.

## 1. Introduction

It is currently well established that cochlear implantation should be as soft and atraumatic as possible, in order to protect the cochlear sensory–neural interface for a better electric stimulation, reduce electrode insertion-induced fibrosis, prevent vestibular deficit, and preserve the cochlea for a possible future implantation, particularly in children [1,2,3,4,5]. In addition, it has been proven that electrode placement into the scala tympani was associated with better speech recognition scores than cases where there was partial or complete electrode migration into the scala vestibuli [2,3]. Also, elevation of the basilar membrane during cochlear implantation resulted in a concomitant drop in cochlear microphonics during real-time electrocochleography recording [6]. Therefore, minimizing insertional trauma is essential for residual hearing preservation and post-implantation hearing outcomes [7,8]—hence the importance of improving electrode array placement techniques, such as using robotic insertion.

Robotic surgery in cochlear implantation has emerged mainly in the second decade of the 21st century, whether to achieve a minimally invasive surgical approach [9,10] or to perform robotic-assisted electrode array insertion inside the cochlea [11,12,13]. To our knowledge, the first robotic electrode insertion in patients was performed in 2019, using the RobOtol^®^ (RobOtol, Collin Medical, Bagneux, France), and reported in 2021 [12]. Since then, around 1500 patients worldwide have benefited from this innovative technique by means of the RobOtol^®^. Some studies have been conducted with the RobOtol^®^ on cadaveric temporal bones (TBs) [14,15] and others have been clinically conducted on robotic electrode insertion in patients [16,17,18]. Our objective was to evaluate manual placement of cochlear implant electrodes and robot-assisted placement using the RobOtol^®^ on human TBs in terms of endocochlear trauma and completion of insertion. Results were analyzed by means of micro-computed tomography (micro-CT) imaging.

## 2. Materials and Methods

Temporal bones were harvested from humans who had given their written consent for body donation prior to their death. All body donations met the requirements of the Declaration of Helsinki on the Ethical Use of Human Material and the bio-ethic laws of our country (Ethical Committee referral file N°42-23-06). TBs were fixed in diluted formalin (3% concentration) and refrigerated at 4 °C for a short period of time before dissection.

**Figure 1:**
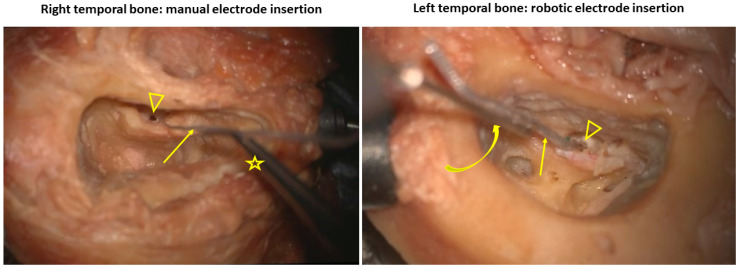
Comparison of insertion of the electrode array (straight arrows) in the same body specimen: right temporal bone manually using the Medel forceps (star) and left temporal bone using the robotic arm and specific inserter (curved arrow). Arrowhead: round window opening.

A canal-wall-up mastoidectomy followed by posterior tympanotomy (facial recess approach) was performed, and the round window (RW) membrane was fully exposed by drilling the posterosuperior bony overhang of the RW niche. We used a surgical microscope (OPMI Pico Technoscope with a table console attachment, Carl Zeiss AG, Oberkochen, Germany) and a surgical microdrill (Midas Rex Legend Stylus Drill, Medtronic, Minneapolis, MN, USA). Then a cochlear implantation was performed through the RW membrane. The electrode array used for cadaveric use was the same for all specimens, SYNCHRONY FLEX24 from MEDEL (Medel Elektronische Geräte, GmbH, Innsbruck, Austria), which is a 24 mm straight and flexible electrode array (active stimulation segment 20.9 mm, 0.5 mm diameter at its tip and 0.8 mm at its base) containing 12 electrode contacts made of platinum and a silicone array containing metallic conducting leads. The right TB was manually implanted at a mean insertion speed of 1 mm/s, while the left TB *of the same body* was implanted using robot-assisted insertion with the active arm of the RobOtol^®^ and the inserter dedicated to MEDEL electrodes (RBT 2306) at a constant insertion speed of 0.3 mm/s (Figure 1). The robot details are the following: fully dedicated to otologic surgery, version V4+, software 3.2.4, 2nd-generation active (automatized) arm, thrust control of speed 0.1–1 mm/s; we used a robot model dedicated to research. In both groups, electrode insertion was interrupted when 1st resistance was noticed: felt by the surgeon in manual implantation on the right and when there was a beginning of electrode array kinking or deformation in robot-assisted implantation on the left. All cochlear implantations, whether manual or robot-assisted, were carried out by the same experienced surgeon (A.K.).

Thereafter, implanted TBs were trimmed to be reduced to a size less than 5 × 5 × 5 cm in order to fit into the tunnel of the micro-CT; care was taken so that the cutting saw did not cross the cochlea. The micro-CT used is a high-resolution microscopic tomograph VivaCT-40 (Scanco Medical, Brüttisellen, Switzerland) that is available in our research laboratory. We defined the following acquisition setup for μCT according to our protocol [5]: scout = 90°, tube voltage = 70 kVp, beam current = 114 µA, field of view (FOV) = 30.7 mm, time of acquisition = 57.8 mn, and number of slices = 1056. The microtomographic scans provided high-resolution images with a nominal non-isotropic voxel size of 15 μm and a slice increment of 15 μm thickness. The relevant images were separately interpreted by 2 otologists (A.K. and K.D.), who looked for any possible trauma inside the cochlea or damage to the implanted electrode array and also evaluated the number of electrodes which remained extracochlear. No statistical analysis was performed, given the limited number of TBs; we favored a descriptive analysis of micro-CT images rather than a statistical comparative study. We used the classification of cochlear trauma described by Eshraghi et al. (2003): (1) elevation of basilar membrane; (2) rupture of basilar membrane; (3) electrode in scala vestibuli; (4) severe trauma such as fracture of osseous spiral lamina or modiolus or tear of stria vascularis [19]. We also included any possible trauma inflicted to the electrode array such as rupture, kinking, or fold-over.

## 3. Results

**Figure 2:**
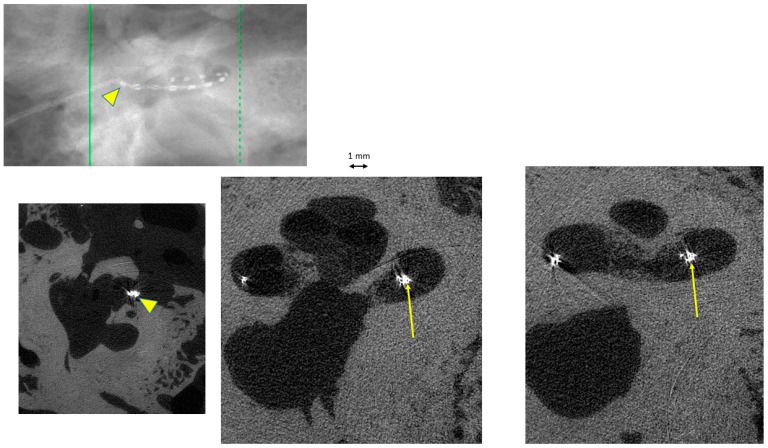
Temporal bone (TB)#25-3 right. Manual insertion of electrodes, with 1 electrode extracochlear (arrowheads). Trauma: elevation of the basilar membrane at the basal turn (arrows).

**Figure 3:**
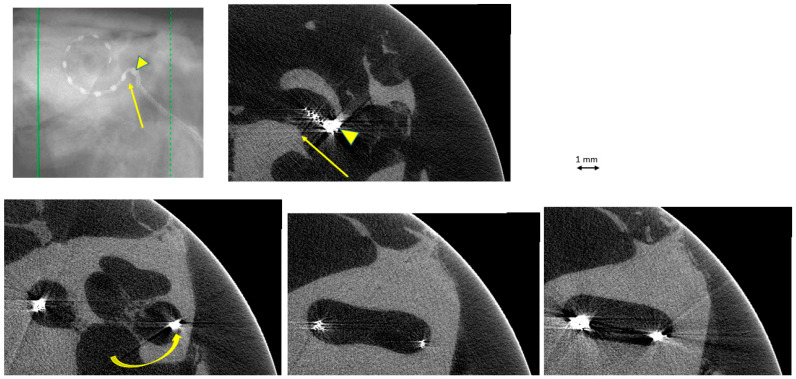
TB#24-7 right. Manual insertion of electrodes, with 1 electrode extracochlear (arrowheads), probably due to a prominent crista fenestrae (straight arrows). Trauma: elevation of the basilar membrane at the basal turn (curved arrow).

**Figure 4:**
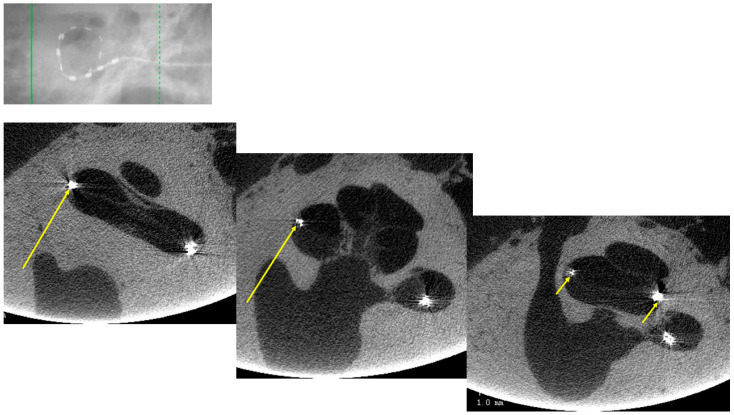
TB#24-5 left. Robot-assisted insertion of electrodes. All electrodes were intracochlear. Trauma: elevation of basilar membrane at the pars ascendens (long arrows) and the middle turn (short arrows).

**Figure 5:**
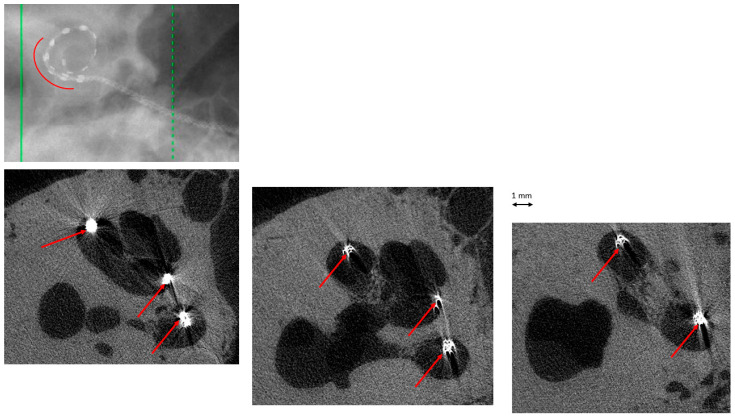
TB#25-4 left. Robot-assisted insertion of electrodes. All electrodes were intracochlear. Trauma: translocation of all electrodes into scala vestibuli (curved line and arrows).

Twenty TBs were implanted (10 subjects), but only sixteen were exploitable. In the remaining four TBs, the cochlea was partially damaged or the electrode array was displaced during the trimming process on the right side and thus could not be compared with the intact left side. There was complete agreement on image interpretation by the two otologists. In the group “manual implantation” of the eight right TBs, there were two cases (25%) of implantation trauma: both cases of elevation of the basilar membrane at the basal turn (Eshraghi stage 1) (Figure 2 and Figure 3). In the group “robotic implantation” of the eight left TBs, there were two cases (25%) of implantation trauma: one case of elevation of the basilar membrane in the pars ascendens and the middle turn (Eshraghi stage 1) (Figure 4) and one case of dislocation of all electrodes in the scala vestibuli (Eshraghi stage 3) (Figure 5) (Table 1). Regarding the completion of electrode insertion, there were six cases (75%) of incomplete insertion in the group “manual implantation” (Figure 2 and Figure 3) and four cases (50%) of incomplete insertion in the group “robotic implantation”. In more detail, in the group “manual implantation”, there were one extracochlear electrode in three TBs, two extracochlear electrodes in two TBs, and three extracochlear electrodes in one TB. In the group “robotic implantation”, there were one extracochlear electrode in three TBs and four extracochlear electrodes in one TB (Table 2).

## 4. Discussion

Our study described possible trauma secondary to cochlear implantation, using robot-assisted electrode insertion and manual insertion. Results were fairly similar, despite the absence of statistical analysis, given the small sample size. On the other hand, some studies have shown the superiority of robotic insertion. In a cadaveric study on 20 TBs, and using a RobOtol^®^ prototype, Torres et al. (2018) showed that robot-assisted electrode insertion yielded a 3-fold slower and constant speed of insertion and a lower rate of dislocation into the scala vestibuli than manual insertion, with the latter being faster and saccadic [15]. However, in contrast to the surgeon’s hands, the RobOtol^®^ lacks haptic feedback. Furthermore, the RobOtol^®^ has 6 degrees of freedom (DOFs), while the human wrist–hand complex has 27 DOFs [20]. In another TB study, Torres et al. (2017) defined an optimal electrode insertion axis, which is the closest axis to the scala tympani centerline avoiding the facial nerve, and compared four techniques: robotic implantation (RobOtol^®^ prototype) and manual implantation, each one with and without image-guidance [14]. The alignment error (angle in degrees) between the insertion tool and the predefined optimal axis was lower in the robotic group, compared to the manual group, whether or not combined with navigation.

It is known that robotic introduction of electrodes inside the cochlea can be set at a speed that is much slower and more constant than manual implantation. Some works have focused on the benefit of this slow insertion. In an experimental study on an artificial scala tympani model, Kontorinis et al. (2011) found that the higher the speed of electrode array insertion inside the model is, the greater the insertions forces are [21]. In a clinical study on 40 patients divided into two groups [first standard electrode insertion (60 mm/s) and second slow electrode insertion (15 mm/s)], Rajan et al. (2013) noted that in the second group (slow insertion), there was a higher rate of complete electrode insertion and preservation of residual hearing, as well as a lesser rate of balance disturbances [22]. Using a cochlear model, Todt el al. (2016) noticed that at a constant speed of electrode insertion of 0.2 mm/s, the maximum amplitude in pressure changes decreased by 20-fold from free-hand insertion to fully automatic insertion. This is thus in favor of maximum stability and support during electrode insertion in order to avoid micromovement-related pressure changes [23].

Three clinical studies comparing manual and robotic electrode placement are summarized here and seem to be in favor of robotic insertion. Daoudi et al. (2021) found that the rate of scalar translocation of the straight electrodes was lower with the RobOtol^®^ than with manual insertion [16]. Rates were higher in both groups using the precurved electrodes. Gersdorff et al. (2025) compared two groups of patients who were all implanted with the same type of straight electrode [17]. Results were better in the group with robotic insertion (RobOtol^®^) in terms of apical electrode impedance declining with time, preservation of residual hearing, and speech discrimination improvement. Khan et al. (2025) analyzed hearing preservation in patients undergoing cochlear implantation with straight electrodes, either manually or robotically assisted using the iotaSOFT system (iotaMotion Inc., Iowa City, IA, USA) [18]. There was a slight tendency in favor of the “robotic group” compared to the “manual group”, but the difference did not find reach statistical significance.

Another much larger-scale clinical, prospective, randomized study has been conducted across several academic centers in France using the RobOtol^®^, and patient inclusions were closed on 31 December 2025. Results will be presented in 2026.

Regarding the completion of electrode introduction in the cochlea, the anatomical relevance of incomplete insertion is that the higher the number of extracochlear electrodes is, the shorter the remaining electrode array inside the cochlea is. Consequently, more apical sites within the cochlea coding for low frequencies will not be stimulated. Clinically, there is not enough evidence as to whether or not *limited* incomplete electrode insertion in the cochlea is associated with less favorable hearing outcomes [24,25].

Our work has three limitations. First, the number of analyzed specimens is limited. In fact, we implanted 20 TBs and ended up with 16. That being said, it is currently difficult to have a greater number of human TBs given ethical issues and impediments. In addition, the procedure needed to obtain the definitive results is tedious and time-consuming. Indeed, every TB needed to be extracted from the head, dissected (posterior tympanotomy), implanted, sectioned, and imaged by micro-CT. The procedure also required the availability of the RobOtol^®^ prototype dedicated to research. The second limitation is that, being a study on cadavers, a certain degree of pre-existing damage to cochlear microstructures might occur in TBs, spontaneously (postmortem) or during the conservation or trimming process, prior to cochlear implantation. Consequently, this could create some bias in the interpretation of cochlear trauma following electrode insertion, particularly regarding the basilar membrane in our study. This being said, we only interpreted basilar membrane elevation when there was contact between an electrode and the basilar membrane, i.e., when the electrode caused a lift, a deformation, or a tenting of the basilar membrane.

In addition, the anatomical results obtained in cadaveric TBs do not necessarily reflect auditory results or vestibular sequelae clinically. That being so, and as mentioned earlier, it is generally known that less traumatic electrode introduction inside the cochlea is associated with a better rate of residual hearing preservation [22,26], higher speech discrimination scores [3], and probably preservation of vestibular function [22]; hence, atraumatic cochlear implantation should always be targeted. Therefore, the authors still use the RobOtol^®^ for electrode array insertion and are testing new techniques to optimize electrode introduction, such as an image-guidance hand piece coupled to the active arm of the robot to target the optimal axis of insertion. The third limitation is that in 10 of the 16 specimens, one to four basal electrodes remained outside the cochlea. This is probably due to the absence of perilymph inside the cochlea and thus to increased friction forces, but also to the conservation methods of the TBs (fixation and refrigeration), and finally to the fact that the authors stopped insertion at the point of first resistance in order to achieve non-traumatic implantation to the cochlea and the electrodes.

On another note, although we had previously proven the reliability of micro-CT for the analysis of cochlear microstructures [5], a technical detail that we are still improving is the visualization of basilar membrane elevation by the electrodes, which has been relatively difficult and demanded the analysis and interpretation of a great number of images for each implanted TB.

## 5. Conclusions

We have conducted a human cadaveric temporal bone study using manual and robot-assisted (RobOtol^®^) electrode insertion and analyzed cochlear microtraumas through micro-CT imaging. Both techniques yielded fairly similar results, in terms of endocochlear trauma and completion of electrode insertion. New temporal bone and clinical studies of a larger scale are needed to determine the possible benefit of robot-assisted electrode insertion in patients undergoing cochlear implantation.

## Figures and Tables

**Table 1:** Interpretation of results on micro-CT scans after manual and robotic insertion of electrodes in terms of cochlear/electrode array trauma. TB: temporal bone; BM: basilar membrane; SV: scala vestibuli.

Body (8 in Total)	Right TB (Manual Insertion)	Left TB (Robotic Insertion)
25-1	No trauma	No trauma
25-3	Trauma: BM elevation	No trauma
25-4	No trauma	Trauma: migration in SV
25-5	No trauma	No trauma
24-2	No trauma	No trauma
24-5	No trauma	Trauma: BM elevation
24-7	Trauma: BM elevation	No trauma
24-12	No trauma	No trauma

**Table 2:** Interpretation of results on micro-CT scans after manual and robotic insertion of electrodes in terms of completion of electrode insertion. TB: temporal bone. EE: extracochlear electrode(s).

Body (8 in Total)	Right TB (Manual Insertion)	Left TB (Robotic Insertion)
25-1	Incomplete insertion (1 EE)	Incomplete insertion (1 EE)
25-3	Incomplete insertion (1 EE)	Incomplete insertion (1 EE)
25-4	Incomplete insertion (3 EE)	Complete insertion
25-5	Complete insertion	Complete insertion
24-2	Incomplete insertion (2 EE)	Incomplete insertion (4 EE)
24-5	Incomplete insertion (2 EE)	Complete insertion
24-7	Incomplete insertion (1 EE)	Incomplete insertion (1 EE)
24-12	Complete insertion	Complete insertion

## Data Availability

The data presented in this study are available on request from the corresponding author due to privacy.
